# Immunological mechanisms and emerging therapeutic targets in alcohol-associated liver disease

**DOI:** 10.1038/s41423-025-01291-w

**Published:** 2025-05-21

**Authors:** Haiyuan Shen, Suthat Liangpunsakul, Yasuko Iwakiri, Gyongyi Szabo, Hua Wang

**Affiliations:** 1 Department of Oncology, The First Affiliated Hospital of Anhui Medical University, Hefei, China; 2 The Key Laboratory of Anti-inflammatory and Immune Medicine, Ministry of Education, Anhui Medical University, Hefei, China; 3 Division of Gastroenterology and Hepatology, Department of Medicine, Indiana University School of Medicine, Indianapolis, IN USA; 4 Department of Biochemistry and Molecular Biology, Indiana University School of Medicine, Indianapolis, IN USA; 5 Roudebush Veterans Administration Medical Center, Indianapolis, IN USA; 6 Section of Digestive Diseases, Department of Internal Medicine, Yale University School of Medicine, New Haven, CT USA; 7 Department of Medicine, Division of Gastroenterology, Beth Israel Deaconess Medical Center, Harvard Medical School, Boston, MA USA

**Keywords:** Alcohol-associated liver disease, Alcohol-associated hepatitis, Inflammation, Immune cells, Gut-liver axis, Mechanisms of disease, Inflammation

## Abstract

Alcohol-associated liver disease (ALD) is a major global health challenge, with inflammation playing a central role in its progression. As inflammation emerges as a critical therapeutic target, ongoing research aims to unravel its underlying mechanisms. This review explores the immunological pathways of ALD, highlighting the roles of immune cells and their inflammatory mediators in disease onset and progression. We also examine the complex interactions between inflammatory cells and non-parenchymal liver cells, as well as their crosstalk with extra-hepatic organs, including the gut, adipose tissue, and nervous system. Furthermore, we summarize current clinical research on anti-inflammatory therapies and discuss promising therapeutic targets. Given the heterogeneity of ALD-associated inflammation, we emphasize the need for precision medicine to optimize treatment strategies and improve patient outcomes.

## Introduction

The widespread misuse of alcohol poses a major global health threat, leading to profound social, economic, and medical consequences. Rising alcohol consumption has been accompanied by a significant increase in disease burden and mortality rates worldwide [1, 2]. According to the World Health Organization, nearly 75 million people suffer from alcohol-related conditions, with ~3 million deaths annually attributed to alcohol consumption [3]. Furthermore, global alcohol intake continues to rise, with projections estimating an increase in average per capita consumption from 8 liters to 8.4 liters by 2025 [4, 5].

The liver is particularly vulnerable to alcohol-induced damage due to its central role in metabolizing alcohol and filtering toxins from the bloodstream [6, 7]. The byproducts of alcohol metabolism can cause significant liver injury, leading to alcohol-associated liver disease (ALD) [8, 9]. ALD encompasses a spectrum of conditions, ranging from simple steatosis to more severe forms such as steatohepatitis, cirrhosis, and fibrosis, ultimately progressing to hepatocellular carcinoma [10]. Among these, severe alcohol-associated hepatitis (sAH) is the most life-threatening, with 28-day and 90-day mortality rates of 20% and 30%, respectively [11, 12]. Despite advances in basic and translational research, the molecular mechanisms driving AH pathogenesis remain largely unclear.

Despite growing public awareness of alcohol-induced liver toxicity, alcohol consumption continues to rise [1, 13]. Currently, the U.S. Food and Drug Administration has not approved any pharmaceutical treatments for ALD, underscoring the urgent need for effective therapies [14]. While the precise pathophysiological mechanisms of ALD remain incompletely understood, strong evidence links inflammation to its progression. In ALD, liver inflammation results from both direct and indirect hepatic injury caused by excessive alcohol intake. Ethanol, acetaldehyde, and lipopolysaccharide (LPS) activate inflammatory cascades that drive alcohol-induced liver damage [15]. Although inflammation is a protective response to harmful stimuli, it also plays a central role in ALD pathogenesis [16, 17]. This review explores the role of inflammation in ALD development and progression and examines current and emerging therapeutic targets aimed at mitigating inflammation in this disease.

## Causes and progression factors of liver inflammation

### Direct damage caused by alcohol metabolites

Ethanol metabolism in the liver occurs through three primary pathways: the alcohol dehydrogenase (ADH) pathway in the hepatocyte cytosol, the cytochrome P450 2E1 (CYP2E1) pathway in the endoplasmic reticulum, and the catalase (CAT) pathway in peroxisomes [18]. These processes convert ethanol into the toxic intermediate acetaldehyde [2, 19]. Acetaldehyde is further metabolized into acetate by mitochondrial acetaldehyde dehydrogenase 2 (ALDH2) and cytosolic aldehyde dehydrogenase (ALDH1) [18]. The resulting acetate is released into the bloodstream and oxidized to carbon dioxide in extrahepatic tissues [7, 19]. Ethanol disrupts mitochondrial respiratory function, leading to structural and functional impairments [20]. Acetaldehyde is even more toxic than ethanol due to its high reactivity, forming covalent adducts with proteins [21, 22], phospholipids [23], and nucleic acids [24], thereby altering their biological functions. Notably, a recent study revealed that a significant portion of acetaldehyde produced in the liver is excreted into the intestine via bile, where it is further metabolized by intestinal epithelial ALDH2 [25]. This finding suggests that targeting ALDH2 in both the liver and intestine may offer a novel therapeutic approach for ALD.

Chronic alcohol consumption leads to increased hepatic levels of CYP2E1 [18, 26]. While enhanced alcohol metabolism may seem protective, elevated CYP2E1 accelerates ethanol oxidation, resulting in higher acetaldehyde production and excessive reactive oxygen species (ROS) generation, including hydroxyethyl radicals, superoxide anions, and hydroxyl radicals [27, 28]. The accumulation of ROS damages mitochondrial DNA and proteins, leading to glutathione depletion [29]. Notably, ROS can react with unsaturated lipids to form lipid peroxides such as malondialdehyde, 4-hydroxy-2-nonenal (4-HNE), and acrolein, further exacerbating oxidative stress [30, 31]. Hepatocyte injury caused by acetaldehyde and ROS generates damage-associated molecular patterns (DAMPs), which activate inflammatory signaling, drive apoptosis and necrosis, and contribute to ALD progression [32, 33].

### Gut microbial disorders

Ethanol and acetaldehyde can impair the intestinal mucosa and alter gut microbiota composition, facilitating the translocation of pathogen-associated molecular patterns (PAMPs), such as LPS, peptidoglycan, and lipoproteins, from the gut to the liver [34]. Gut-derived antigens induce distinct immune activation in hepatic parenchymal and non-parenchymal cells through engagement of pattern recognition receptors (PRRs), including Toll-like receptors (TLRs) [35, 36], NOD-like receptors (NLRs) [37], and C-type lectin receptors (CLRs) [38]. A prototypical example involves LPS activation of Kupffer cells (KCs) through TLR4 signaling, which promotes the secretion of pro-inflammatory mediators such as CC-chemokine ligand 2 (CCL2), interleukin (IL)-8, tumor necrosis factor-α (TNF-α), IL-1β, and IL-6 [35, 39]. These cytokines and chemokines recruit circulating immune cells into the liver, disrupting immune homeostasis and promoting liver injury [35, 39].

### Activation of immune responses

**Fig. 1:**
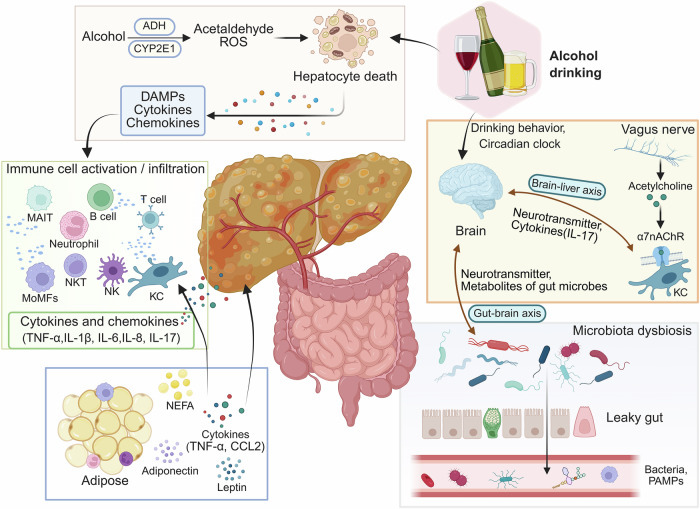
Triggers of Immune Response in ALD. Acetaldehyde and reactive oxygen species (ROS) generated during alcohol metabolism activate inflammatory signaling pathways, leading to hepatocyte injury, apoptosis, and necrosis. These events trigger the release of inflammatory mediators and damage-associated molecular patterns (DAMPs). Chronic alcohol consumption disrupts the intestinal barrier, increasing permeability and facilitating gut-derived microbial products’ translocation to the liver via the portal vein, thereby initiating an immune response. The nervous system modulates hepatic immune cell phenotypes through sympathetic signaling and neurotransmitters. Additionally, adipose-liver crosstalk, mediated by cytokines, adipokines, and metabolic signals, further amplifies immune activation and promotes the release of pro-inflammatory cytokines and chemokines. Collectively, these inter-organ interactions drive liver inflammation, exacerbate hepatocellular damage, and contribute to the progression of ALD

The migration of inflammatory cells and activation of the inflammatory cascade can indirectly contribute to liver injury. The pathogenic cascade is initiated by innate immune activation, characterized by KCs mobilization and neutrophil infiltration [8]. DAMPs released by dying hepatocytes, along with PAMPs, stimulate Kupffer cells to upregulate proinflammatory cytokines, leading to the recruitment of neutrophils and macrophages to the liver [40, 41]. Neutrophil migration into the parenchyma exacerbates hepatocellular damage through ROS generation and proteolytic enzyme release, ultimately contributing to alcohol-induced hepatocyte apoptosis [42]. Protein adducts and neoantigens generated through oxidative stress and lipid peroxidation further provoke an adaptive immune response, characterized by T-cell and B-cell infiltration [8]. Nevertheless, the precise mechanisms underlying adaptive immunity-mediated hepatocellular injury and inflammatory amplification in AH patients remain elusive. Chronic ethanol exposure not only induces hepatocyte death but also suppresses regenerative proliferation, thereby creating a pathological microenvironment conducive to ALD progression [43]. Additionally, liver injury activates hepatic stellate cells (HSCs), which secrete transforming growth factor-β (TGF-β) and collagen, ultimately driving liver fibrosis [44]. (Fig. 1).

## Roles of immune cells

### Macrophages

Macrophages in the liver can be broadly classified into two categories. The first group consists of KCs, the largest resident macrophage population in the liver [45]. They serve as the primary line of defense, clearing pathogens, endotoxins, and damaged cell debris from the bloodstream while also regulating liver immunity [45]. The second category comprises monocyte-derived macrophages (MoMFs), which migrate from the peripheral blood and differentiate into macrophages in response to liver injury or inflammation, playing key roles in immune regulation and tissue repair [46, 47].

Chronic alcohol intake disrupts gut microbiota and compromises intestinal barrier function, allowing bacterial endotoxins such as LPS to enter the bloodstream [48]. LPS binds to Toll-like receptor 4 (TLR4) on the surface of Kupffer cells, activating both the MyD88-dependent pathway, which mediates NF-κB signaling, and the MyD88-independent pathway involving interferon regulatory factor 3 (IRF3), ultimately upregulating TNF-α transcription [49, 50]. Additionally, TLR4 signaling triggers the extracellular signal-regulated kinase and p38 MAPK pathways, further increasing the expression of downstream molecules and promoting the production of TNF-α and other inflammatory cytokines and chemokines [51, 52].

During alcohol exposure, Kupffer cells are a major source of ROS, which contribute to the generation of TNF-α and IL-6 [53]. These inflammatory cytokines production amplifies immune cell activation and recruitment, perpetuating hepatic inflammation. Notably, Kupffer cells also play a critical role in liver regeneration. Through NF-κB and signal transducer and activator of transcription 3 activation, they release TNF-α and IL-6 that reduce hepatocyte apoptosis and promote hepatocyte proliferation, facilitating liver recovery after alcohol-induced injury [54–56]. Given their dual role in inflammation and regeneration, indiscriminate inhibition of Kupffer cells may not be an ideal therapeutic approach for ALD. The endoplasmic reticulum (ER) protein Nogo-B (also known as Reticulon 4B) promotes a pro-inflammatory state, exacerbating liver injury and steatosis [57]. The absence of Nogo-B increases ER stress, which shifts Kupffer cells/macrophages toward an anti-inflammatory phenotype, thereby reducing liver inflammation [57]. Targeting Nogo-B may present a potential therapeutic approach for ALD. In response to hepatocyte injury, Kupffer cells release CCL2, facilitating monocyte recruitment and migration to the liver, where they differentiate into pro-inflammatory macrophages [58]. The chemokine receptors CCR2 and CCR5 mediate interactions among intrahepatic immune cells, promoting the activation and migration of peripheral monocytes into the liver [59, 60]. Consequently, the upregulation of these key receptors is a hallmark of ALD [61]. Given their role in disease progression, modulating macrophage phenotypic transformation and simultaneously inhibiting CCR2 and CCR5 represent promising therapeutic strategies for ALD. However, it is important to note that the recent Phase III Randomized Study on Cenicriviroc (a dual CCR2/CCR5 antagonist) has not demonstrated the efficacy of Cenicriviroc in treating liver fibrosis in adult patients with NASH [62]. Moreover, considering issues such as the safety of the drug, the development of targeted therapies targeting CCR2/CCR5 for the treatment of ALD requires the cautious approach of relevant researchers.

The precise role of MoMFs in ALD remains incompletely understood. MoMFs (CD11b^hi^F4/80^lnt^Ly6C^+^) and KCs (CD11b^low^F4/80^hi^Ly6C^−^) can be distinguished by their differential expression of cell surface markers [47]. Chronic alcohol consumption increases the number of recruited macrophages in the liver, promoting the differentiation of Ly6C^hi^ monocytes into pro-inflammatory macrophages that contribute to tissue damage [47]. Additionally, the phagocytosis of apoptotic hepatocytes enables Ly6C^hi^ monocytes/macrophages to transition into Ly6C^low^ monocytes/macrophages, which subsequently differentiate into tissue-protective macrophages [47]. The balance between these two subpopulations is thought to dictate the functional role of recruited macrophages in ALD pathogenesis [47, 63].

### Neutrophils

Neutrophils play a critical role in the immune system, primarily mediating inflammatory responses. They contribute significantly to tissue repair and immune regulation [64, 65] by coordinating immune and inflammatory processes through phagocytosis, ROS generation, degranulation, cytokine and chemokine production, and the release of neutrophil extracellular traps (NETs) [66–69]. These mechanisms collectively influence alcohol-induced liver injury [66–69]. Both circulating and hepatic neutrophil counts are elevated in patients with AH [70], and the neutrophil-to-lymphocyte ratio correlates with AH-related mortality [71, 72]. A variety of CXC and CC chemokines are significantly upregulated in AH patients compared to healthy individuals, with their expression levels positively associated with neutrophil infiltration and the severity of liver injury [73, 74]. IL-8, a key neutrophil chemoattractant, binds to CXCR1 and CXCR2 receptors on neutrophil surfaces, promoting their chemotaxis and infiltration into the liver [75]. Moreover, liver sinusoidal endothelial cells (LSECs) contribute to neutrophil recruitment by upregulating the expression of adhesion molecules such as E-selectin [76].

The activation of neutrophils in ALD is primarily driven by toxic metabolic byproducts of alcohol metabolism and oxidative stress responses [45, 77, 78]. Upon activation, neutrophils release various inflammatory mediators, including cytokines, chemokines, and ROS, which exacerbate liver inflammation [45, 77, 78]. Alcohol exposure induces neutrophils to release inflammatory cytokines such as TNF-α and IL-1β, while also enhancing the production of additional chemokines, such as IL-8/CXCL8 [75, 79]. Alcohol-induced phosphorylation of the Bruton’s tyrosine kinase plays a key role in neutrophil activation, increased granulopoiesis, and neutrophil-mediated liver damage [80]. NETs, which serve to capture and eliminate pathogens, can contribute to tissue damage and perpetuate inflammatory responses when produced in excessive amounts [78]. Intestinal-derived endotoxins can activate neutrophils via the TLR4 pathway, promoting their migration and NET release [68]. Platelet activation also stimulates NET formation, which plays a key role in liver injury and inflammation [68]. Additionally, histones within NETs can bind to TLR9 on HSCs, activating them and stimulating their proliferation and extracellular matrix synthesis, thus accelerating liver fibrosis [81]. While neutrophils contribute to the progression of ALD, neutrophil-specific miR-223 appears to exert a protective effect against liver injury. Studies have shown that serum miR-223 levels are elevated in patients with AH, and miR-223 deficiency exacerbates ethanol-induced inflammation, oxidative stress, and liver damage [82]. The contrasting effects of neutrophils in ALD may be partly due to the heterogeneity within the neutrophil population [83, 84].

Recent research suggests that neutrophils can be categorized into two subtypes: high-density neutrophils (HDNs) and low-density neutrophils (LDNs) [84]. HDNs exhibit a hyperactivated phenotype, capable of producing more ROS and NETs, which aggravate liver injury [84]. In contrast, LDNs display a functionally exhausted phenotype, with reduced chemotactic and immune functions, impairing their ability to eliminate pathogens and necrotic cells [84]. HDN-derived NETs can promote the retention of LDNs in the liver, preventing their clearance by macrophages. The accumulation of LDNs further suppresses immune responses and increases the risk of infection [84]. Further exploration of neutrophil heterogeneity is essential for gaining a deeper understanding of their role in the pathogenesis of AH.

Recently, a study utilized single-cell RNA sequencing to identify a distinct population of IL-8^+^ neutrophils in human sAH samples [75]. Upon infiltrating the liver, peripheral neutrophils from sAH patients are activated by the inflammatory milieu, including mediators such as TNF-α and IL-1β, leading to elevated expression of IL-8 [75]. The resultant production of IL-8^+^ neutrophils exacerbates systemic inflammation and further recruits additional neutrophils to the liver. This self-perpetuating recruitment of IL-8+ neutrophils in sAH patients may culminate in uncontrolled hepatic inflammation and subsequent liver failure [75]. This significant discovery suggests that targeting IL-8^+^ neutrophils could be a promising therapeutic strategy, potentially mitigating the inflammatory response specific to sAH without impacting other hepatic conditions [75, 85].

### B cells

Acetaldehyde and other intermediate products produced during alcohol metabolism are immunogenic and can bind to proteins in hepatocytes, forming new antigenic epitopes [86]. B cells, as antigen-presenting cells, can recognize and internalize these antigens, process them, and present them to T cells, thereby activating T-cell-mediated immune responses [87, 88]. Antibodies produced by B cells bind to acetaldehyde-protein adducts and other antigens, forming immune complexes [89]. These complexes can deposit on hepatocyte membranes or LSECs, activating the complement system and triggering local inflammatory responses, leading to hepatocyte damage [89]. Ahmadi et al. identified unique antibodies in the liver of sAH, which could not only recognize bacterial (*Escherichia coli*) antigens but also cross-react with numerous human antigens. The deposition of a considerable amount of antibodies, along with complement activation and immune cell activation, might result in acute liver failure due to antibody-mediated inflammation [90].

Due to alcohol-induced inhibition of B lymphocyte differentiation, patients with ALD experience a reduction in B lymphocytes, which are essential for humoral immune responses [91]. Elevated levels of immunoglobulin A in circulation are a characteristic feature of alcohol-related cirrhosis, arising from TLR9 activation in B cells [92]. B cells play a complex, dual role in the onset and progression of ALD. On one hand, B cells contribute to immune surveillance and inflammatory responses in the liver by recognizing antigens and secreting cytokines and antibodies, which facilitate hepatocyte damage and liver fibrosis [92]. On the other hand, IgA synthesized by B cells in the liver plays a crucial role in clearing antigens from the gut and is essential for safeguarding the body against pathogens [93]. Targeting B cells and their associated mechanisms may offer novel therapeutic strategies for treating ALD.

### T cells

Individuals with ALD show significant infiltration and activation of CD3^+^ T cells in hepatic tissue [94]. Circulating T cells exhibit elevated expression of activation markers, such as CD69 and CD38, but there is a notable reduction in interferon-γ production upon stimulation [95, 96]. An extensive immediate Th1 response is observed in ALD patients, which may exacerbate inflammatory responses and contribute to tissue damage [97]. Th17 cells produce interleukin-17 (IL-17), which recruits and activates neutrophils, exacerbating liver inflammation and fibrosis [98]. Additionally, Th17 cells may aid in liver repair by producing more IL-22 [99]. The number of gut microbiota-specific Th17 cells, such as those specific to Candida albicans, increases during ALD, and these cells migrate to the liver, further amplifying the inflammatory response [100]. The cytokines secreted by Th1 and Th17 cells (e.g., IFN-γ and IL-17) can directly act on hepatic stellate cells, stimulating their activation and proliferation, which leads to the synthesis of extracellular matrix components like collagen and contributes to liver fibrosis [101, 102]. Recent studies have highlighted that CD8^+^ T cells exhibit selective depletion in the duodenum of patients with ALD, resulting in compromised intestinal barrier function and exacerbation of ALD pathology. Conversely, a striking accumulation of CD8^+^ T cells is observed in hepatic tissues. These reciprocal alterations in CD8^+^ T cell distribution suggest that therapeutic interventions targeting cellular survival mechanisms and functional restoration of both intestinal and intrahepatic CD8^+^ T cell populations may represent a novel therapeutic strategy for ALD management [103].

Natural killer T (NKT) cells can recognize lipid antigens, including specific lipid metabolic products generated by the intestinal microbiota [30, 104]. In ALD, dysbiosis of the intestinal microbiota allows these lipid antigens to enter the liver, where they activate NKT cells [105–107]. Type I NKT cells, including invariant NKT cells, are recruited and activated by IL-1β produced by Kupffer cells, and they typically adopt a pro-inflammatory phenotype in ALD [108, 109]. In contrast, type II NKT cells are activated by sulfonamide compounds and can inhibit the activity of type I NKT cells [110]. The cytokine production profile of NKT cells in patients with severe AH is altered, with notable reductions in IL-22 levels [104]. However, in contrast to the rich population of NKT cells observed in murine livers, the relative scarcity of these immune cells in human hepatic tissue underscores the need for deeper investigation into their potential involvement in the pathogenic mechanisms of ALD.

In contrast, the human liver is rich in mucosal-associated invariant T (MAIT) cells, which comprise 20%-50% of intrahepatic T cells [111]. MAIT cells are a unique subset of T cells predominantly located on mucosal surfaces, capable of recognizing specific metabolic products produced by the gut microbiota (e.g., riboflavin derivatives) [112]. These cells present antigens through their invariant T cell receptor (TCR) to the major histocompatibility complex-related protein 1 (MR1) [113]. MAIT cells help restrict bacterial translocation by producing antimicrobial peptides and cytokines (such as IFN-γ) [112]. However, in patients with severe AH, circulating MAIT cells are rapidly depleted, excessively activated, and exhibit impaired antimicrobial and cytotoxic responses, increasing the risk of bacterial infections [114]. Abnormal activation of MAIT cells may lead to excessive production of inflammatory mediators, further exacerbating liver inflammation [115]. Additionally, MAIT cells may influence the activation and fibrotic processes of HSCs by secreting cytokines such as IFN-γ and IL-17A [116]. Nevertheless, the precise role of MAIT cells in ALD remains to be fully understood.

### NK and Type 1 Innate Lymphoid Cells (ILC1s)

Natural killer (NK) cells can inhibit liver fibrosis progression by directly targeting HSCs or by secreting cytokines such as IFN-γ [115, 117]. However, in patients with ALD, the number of NK cells is reduced, and their function is impaired, with alcohol directly hindering the development of CD11b^+^CD27^+^ NK cells [118]. Chronic alcohol consumption leads to a decrease in the hepatic population of conventional NK cells and impairs their cytolytic function, as evidenced by the reduced expression of natural killer group 2, member D (NKG2D), tumor necrosis factor-related apoptosis-inducing ligand (TRAIL), and IFN-γ [119–121]. Recent studies have shown that after prolonged alcohol exposure, NK cells (but not ILC1s) in the livers of mice undergo apoptosis, leading to a predominance of ILC1s in group 1 innate lymphoid cells (ILCs) and the upregulation of IL-17A, which exacerbates inflammation and steatosis in ALD [122]. Restoring the ILC1/NK cell balance through NK cell transfer has been shown to provide significant protection against alcohol-induced steatohepatitis [122]. Further research is needed to fully understand the distinct types of NK cells and their respective roles in the progression of ALD.

## Roles of cytokines and chemokines

### TNF-α

TNF-α is a pro-inflammatory cytokine primarily produced by macrophages and monocytes, playing a critical role in inflammatory and immune responses [123]. In animal models of ALD, serum and hepatic concentrations of TNF-α are significantly elevated in patients with sAH and closely correlate with the extent of liver injury [17]. However, no significant elevation in TNF-α levels was observed in patients with alcohol use disorder (AUD) who exhibited normal serum biochemical profiles [124, 125]. Acetaldehyde and ROS generated during alcohol metabolism can directly stimulate KCs and HSCs, promoting the transcription and secretion of TNF-α [124, 125]. Chronic alcohol consumption compromises intestinal barrier integrity, allowing bacteria and endotoxins to translocate to the liver [48, 126]. These endotoxins activate KCs through the TLR4 signaling pathway, further increasing TNF-α production [127, 128]. TNF-α can activate TAK1, which subsequently triggers MAPKs and IκB kinases (IKKs), leading to the activation of NF-κB and, ultimately, inflammation and hepatocyte apoptosis [127, 128]. Additionally, alcohol-induced oxidative stress exacerbates lipid peroxidation and cell membrane damage, further enhancing TNF-α gene expression by activating NF-κB [129]. The resulting imbalance in the cytokine network contributes to the progression of liver injury.

### IL-1β

The ingestion of alcohol can activate KCs in the liver, which in turn stimulate the NLRP3 inflammasome and caspase-1, promoting the maturation and release of IL-1β [81, 130]. Upon ligand binding, the IL-1 receptor (IL-1R) subunits form oligomers and engage MyD88, activating NF-κB and MAPKs, thereby initiating pro-inflammatory responses [131]. In individuals with ALD, elevated levels of IL-1β, IL-18, and caspase-1 in the liver are positively correlated with the severity of the condition [132]. IL-1β induces the recruitment of invariant natural killer T cells, leading to liver injury through TNF-α production and the recruitment of neutrophils to the liver [108]. Persistent elevation of IL-1β levels following both alcohol exposure and withdrawal can result in sustained liver inflammation and impaired hepatocyte regeneration, suggesting that IL-1β may serve as a promising therapeutic target for ALD [133]. In preclinical animal models, administration of IL-1β inhibitors, including IL-1 receptor antagonists (IL-1RA) or anti-IL-1β antibodies, has been shown to mitigate liver inflammation, steatosis, and fibrosis [134]. Unfortunately, a clinical trial evaluating drugs targeting the IL-1β pathway, such as anakinra, for the treatment of AH, yielded negative results [135].

### IL-8 and CXCL1

Neutrophil migration is a hallmark of ALD, with IL-8 and CXCL1 being the key factors in recruiting neutrophils to liver tissue [66, 75]. Elevated serum and hepatic levels of IL-8 and CXCL1 are directly correlated with the severity and mortality of AH [16]. Research has shown that circulating IL-8 levels are markedly increased in patients with severe AH, serving as superior indicators of short-term mortality compared to traditional prognostic markers [136]. Recent studies have further revealed that neutrophils are the primary source of IL-8 in AH [75]. In ALD, neutrophils are recruited to the liver and release IL-8 through autocrine and paracrine mechanisms, acting as a chemotactic factor that attracts additional neutrophils to the liver, thus perpetuating a vicious cycle [75]. Studies using animal models of alcohol consumption have demonstrated that blocking the IL-8 receptor (CXCR1/2) effectively reduces liver injury, inflammation, and mortality in mice [137, 138]. CXCL1 was significantly upregulated in mice subjected to a high-fat diet (HFD) and ethanol exposure, while both CXCL1 knockout and anti-CXCL1 treatment protected these mice from neutrophil-mediated liver injury [66]. Consequently, inhibiting IL-8/CXCL1 to reduce neutrophil migration to the liver may represent a promising therapeutic strategy for AH.

### IL-17A

Studies have shown that individuals with AH and cirrhosis exhibit significantly higher levels of IL-17A [98, 139]. IL-17A has the notable ability to stimulate the expression of a broad range of pro-inflammatory cytokines and chemokines, including IL-6, TNF-α, and IL-8, which further amplify the inflammatory response within the liver [98]. The elevation of these factors leads to the recruitment and activation of neutrophils, thereby exacerbating hepatocyte damage. Additionally, IL-17A stimulates the proliferation and collagen synthesis of HSCs and upregulates the expression of matrix metalloproteinases (MMPs), influencing extracellular matrix remodeling and accelerating the progression of liver fibrosis [140].

### IL-6

IL-6 is a multifunctional cytokine produced by immune cells and hepatocytes [141]. Studies have shown that serum IL-6 levels are significantly elevated in patients with ALD and are directly linked to liver dysfunction and the severity of ALD complications [142, 143]. Recent research highlights IL-6’s diverse biological effects, demonstrating its role in inducing the expression of various pro-inflammatory cytokines and chemokines, as well as regulating anti-apoptotic gene transcription and liver regeneration [144]. In mouse hepatocytes, IL-6-mediated activation of the JAK/PI3K/Akt signaling pathway has been shown to inhibit hepatocyte apoptosis [145]. Furthermore, IL-6 activates mtDNA repair enzymes and repair mitochondrial DNA damage in liver cells following chronic alcohol consumption, promoting hepatic cell regeneration and repair [146]. These findings suggest that further investigation into the mechanisms by which IL-6 influences ALD is necessary. Moreover, intervention strategies targeting IL-6 may produce varying outcomes depending on the disease’s inflammatory stage.

### IL-22

IL-22 is a cytokine with hepatoprotective properties, primarily produced by immune cells, including helper T, NK, and NKT cell subsets [147, 148]. Upon binding to its receptor complex, IL-22 activates the JAK/STAT signaling pathway, promoting hepatocyte regeneration and repair [149, 150]. In various acute and chronic liver injury models in mice, IL-22 has been shown to reduce hepatocyte damage and enhance liver regeneration by upregulating the expression of antioxidant and anti-apoptotic genes, such as heme oxygenase-1 (HO-1) and B-cell lymphoma-extra large (Bcl-xL) [151]. In mouse models, ethanol-induced intestinal dysbiosis and reduced levels of associated metabolites lead to decreased intestinal IL-22 expression [152]. This reduction results in diminished intestinal REG3G (Regenerating Family Member 3 Gamma) expression and increased bacterial translocation to the liver, exacerbating liver inflammation [152]. Given that IL-22 receptor expression is limited to epithelial cells and not detectable in immune cells [151], this specificity is expected to reduce potential side effects of IL-22 in clinical applications, making it a promising therapeutic target for ALD [153].

### Other cytokines and chemokines

CCL20 is one of the most significantly upregulated chemokines in patients with AH [73] and is associated with endotoxemia, liver fibrosis, and short-term mortality [154]. Recent RNA sequencing and ELISA findings have shown that several neutrophil chemokines, such as CXCL1, CXCL5, and CXCL6, are also upregulated in severe AH livers. Notably, single-cell RNA sequencing (scRNA-seq) analysis of severe AH livers revealed that hepatic stellate cells produce CXCL5, while hepatocytes express CXCL6 [75]. Serum CCL2 levels have a significant correlation with liver cirrhosis scores in both animal models and clinical patient populations [155]. CCL2 knockout mice show protection against alcohol-induced liver injury by inhibiting inflammatory cytokine production and enhancing lipid oxidation [156].

IL-10 is a potent anti-inflammatory cytokine produced by immune cells, such as macrophages and NK cells, following alcohol consumption or LPS stimulation [157, 158]. IL-10 suppresses the secretion of inflammatory cytokines by macrophages and monocytes [159] and reduces the antigen-presenting capacity of B cells and dendritic cells [158]. Additionally, IL-10 promotes liver regeneration and mitigates liver injury through activation of the JAK-STAT signaling pathway and its downstream effectors [160]. Preclinical research has demonstrated that IL-10 deficiency exacerbates hepatic inflammation and hepatocellular injury in mice, while stimulating IL-10 production in HSCs and KCs can prevent alcohol-induced liver damage [161, 162]. Therefore, IL-10 represents a promising therapeutic target for ALD.

## Interactions of hepatocytes or nonparenchymal cells with immune cells

### Hepatocytes and hepatokines

Hepatocytes play a significant role in shaping the immune environment in ALD. Damaged hepatocytes release a variety of chemokines [163, 164] and DAMPs [165, 166], which promote immune cell infiltration in ALD. Research has shown that alcohol stimulates hepatocytes to release extracellular vesicles containing CD40L in a caspase-3-dependent manner [167], thereby activating macrophages and increasing the production of inflammatory cytokines. Macrophage migration inhibitory factor (MIF), a pleiotropic cytokine/chemokine that signals through interactions with the CD74 receptor and its auxiliary receptors CXCR2, CXCR4, and CXCR7 [168, 169], is elevated in ALD patients [170] and in mice chronically exposed to ethanol [171]. MIF released from injured hepatocytes may act as a DAMP during ALD progression, leading to the recruitment of innate immune cells to the liver and the activation of inflammatory pathways. Targeting MIF release or signaling could offer a viable therapeutic strategy for ALD. Additionally, damaged hepatocytes can produce neutrophil chemotactic factors [172], which are significantly correlated with mortality in AH patients. Notably, miR-223, which is specifically released by neutrophils, has a protective effect on hepatocytes. Neutrophils recruited to the liver release proteases, ROS, and NETs, which are the primary mechanisms underlying liver injury [84, 173].

Hepatocytes are capable of synthesizing and secreting various biological signaling molecules, collectively known as hepatokines. Hepatocyte-derived growth differentiation factor 15 (GDF15) exerts multifaceted effects on ALD [174]. GDF15 regulates the metabolic pathways of macrophages, acquiring an anti-inflammatory function dependent on oxidative phosphorylation, reducing the expression of pro-inflammatory factors, and decreasing the infiltration of monocytes and neutrophils into the liver, thereby alleviating liver inflammation [174, 175]. Leukocyte Cell Derived Chemotaxin-2 (LECT2), which is elevated in patients with metabolic dysfunction-associated steatohepatitis (MASH), selectively promotes JNK phosphorylation in KCs, leading to liver inflammation [176]. Our recent work indicates that LECT2 levels are significantly increased in the serum of AH patients and that LECT2, produced by stressed hepatocytes, plays a crucial role in regulating the inflammatory phenotype of ALD. LECT2 inhibition ameliorates neutrophil NET formation and reduces liver injury in animal models [177]. Thus, targeting hepatokines to modulate inflammation represents a potential therapeutic approach.

### Liver sinusoidal endothelial cells (LSECs)

LSECs play a crucial role in maintaining liver homeostasis and regulating hepatic microcirculation [178]. In response to inflammatory signals, these endothelial cells activate the NF-κB signaling pathway, increasing the expression of inflammatory mediators and adhesion molecules [179]. The chemokines they release, such as CXCL1 and CXCL2, attract monocytes and neutrophils to the liver, where they contribute to the localization and amplification of inflammatory responses [180]. During prolonged alcohol consumption, the expression of intercellular adhesion molecule-1 (ICAM-1) on LSECs is upregulated [173, 181]. This upregulation facilitates the binding of ICAM-1 to the integrin CD11/CD18 complex on neutrophils, thereby enhancing leukocyte adhesion [173, 181]. E-selectin levels are elevated in the livers of both mice and humans with ALD [76, 182]. In models of chronic and binge ethanol exposure, the ablation of E-selectin reduces hepatic neutrophil infiltration and liver injury [76]. LSECs also influence macrophage phenotypes through paracrine signaling of IL-10 and TGF-β [183]. They are a critical component of the liver’s immunosuppressive microenvironment, which dampens KC responsiveness to gut-derived toxins. Hepatic endothelial cells thus play a pivotal role in modulating inflammatory responses in ALD by regulating the recruitment, activation, and adhesion of inflammatory cells.

### Hepatic stellate cells (HSCs)

HSCs exert significant influence over ALD by secreting cytokines such as TGF-β, PDGF, and TIMP-1 [45]. These cytokines orchestrate the recruitment, activation, and fibrotic processes of inflammatory cells, engaging signaling pathways like NF-κB and Smad, along with oxidative stress mechanisms and cellular interactions [184, 185]. Activated HSCs promote macrophage activation and migration by releasing IL-6 and CCL2 [186, 187], while IL-1 and TNF-α secreted by macrophages enhance HSC viability through the activation of NF-κB signaling [188]. Neutrophils also contribute to HSC activation by producing ROS in vitro, and chemokines released by activated HSCs help attract neutrophils to the liver [137, 189]. Interactions between HSCs and various cell types create a positive feedback loop that is closely associated with alcohol-induced liver fibrosis.

## Crosstalk between extrahepatic organs and inflammation within the liver

### Gut and gut microbiota

Research on the role of the microbiota in ALD dates back to 1995. Studies have demonstrated that intestinal decontamination with polymyxin B and neomycin significantly reduces levels of LPS, aspartate aminotransferase (AST), and liver histopathological scores in male Wistar rats that were given alcohol via gavage for 3 weeks [190]. Alcohol administration in mice in the NIAAA model resulted in a significant reduction in the genus *Akkermansia* [191] and oral administration of *Akkermansia muciphila* in the Lieber-DeCarli model ameliorated features of ALD [192]. In mice fed the Lieber–DeCarli alcohol liquid diet for 6 weeks, chronic alcohol consumption reduced the abundance of *Bacteroidetes* and *Firmicutes* [193]. These changes in the gut microbiota were associated with increased plasma endotoxins and liver inflammation [194]. Similarly, over half of patients with alcohol misuse exhibit intestinal barrier dysfunction and dysbiosis [195], with patients with AUD also showing gut microbiota dysbiosis and elevated serum endotoxins of intestinal origin [196, 197]. The abundance of *Enterococcus faecalis* and *Candida* in the gut of patients with AH is strongly correlated with liver disease severity and mortality [198]. In general, gut microbiota dysbiosis leads to increased systemic inflammatory mediators, ammonia, endotoxemia, enhanced intestinal permeability, and alcohol craving [199]. Thus, understanding which aspects of gut microbiota are dysregulated after alcohol consumption and whether these changes influence the progression of ALD could be critical for developing prevention or treatment strategies for ALD [193].

Alcohol-induced dysbiosis primarily affects intestinal permeability and immune cell function through microbial components (such as LPS, peptidoglycan, flagellin, cytolysin, and β-glucan) and metabolic products (including bile acids, short-chain fatty acids (SCFAs), indole derivatives, and vitamin B), thereby exacerbating liver inflammation [200–204]. Peptidoglycan not only stimulates TLR2 receptors on lymphocytes and monocytes but also interacts with NOD2 and NLRP3 [205]. β-glucan, a cell wall polysaccharide found in most fungi, including *Candida* species, circulates to the liver at elevated concentrations and induces the production of mature IL-1β via C-type lectin domain family 7 member A (CLEC7A) on KCs [206]. The reduced abundance of beneficial gut microbiota results in decreased SCFA production, which, in conjunction with alcohol, increases intestinal permeability and allows more PAMPs to enter the liver [207]. Some bile acids bind to the G protein-coupled receptor TGR5 and inhibit TNF production by Kupffer cells via the TGR5-cAMP-dependent pathway. This interaction between bile acids and TGR5 reduces NLRP3 inflammasome activation, inhibits NF-κB signaling, and induces IL-10 production via CREB [208]. Indole-3-acetic acid (IAA) promotes the expression of IL-22 and Reg3γ through the aryl hydrocarbon receptor (AHR), enhancing intestinal mucosal integrity and mitigating alcohol-induced liver inflammation [209].

**Fig. 2:**
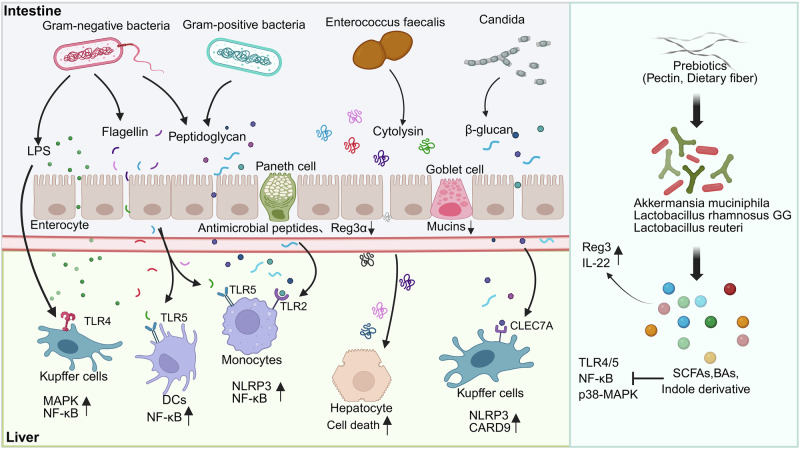
Interaction Between Gut Microbiome and both Hepatocytes and Hepatic Immune Cells. Chronic alcohol consumption and gut dysbiosis synergistically impair intestinal barrier integrity through suppressing the expression of critical defense factors, including antimicrobial peptides, regenerating islet-derived protein 3α (REG3α), and mucins, thereby exacerbating pathological permeability in the gut-liver axis. Gut microbial components, including pathogen-associated molecular patterns (PAMPs) such as lipopolysaccharide, flagellin, peptidoglycan, cytolysin, and β-glucan, can translocate across a compromised intestinal barrier and activate inflammatory signaling pathways in hepatic immune cells, particularly Kupffer cells. This activation triggers an inflammatory cascade that contributes to liver injury and disease progression. Conversely, prebiotics and probiotics may exert hepatoprotective effects by strengthening gut barrier integrity and modulating immune responses through their metabolites, potentially mitigating inflammation and liver damage

In addition to fecal microbiota transplantation, other interventions aim to modify the gut microbiome composition through diet or bacteriophage therapy. Modulation of the gut microbiota in ALD mouse models using the prebiotic pectin has been shown to alter the gut microbiome and metabolome of alcohol-fed mice [210]. This intervention results in enhanced tryptophan metabolite production and a reduction in liver injury and inflammation following microbial transplantation [210]. Recent studies have demonstrated that soluble dietary fiber can alleviate alcohol-induced liver injury in mice by regulating conjugated bile acid levels in the gut through *Bacteroides acidifaciens*, thereby modulating the FXR-FGF15 signaling pathway [211]. Additionally, the application of bacteriophages targeting *Enterococcus faecalis* has been shown to reduce alcohol-induced liver injury in humanized mice, providing a novel approach for the targeted treatment of ALD [198]. In summary, strategies targeting the gut microbiota, such as probiotics, prebiotics, or specific phages, offer promising therapeutic avenues for ALD (Fig. 2).

### Adipose tissue

Adipose tissue is primarily composed of adipocytes [45], witch store excess energy by taking up circulating non-esterified fatty acids (NEFA) and subsequently esterifying them into triglycerides [212]. Long time alcohol consumption significantly triggers adipocyte apoptosis within adipose tissue in mice, enhances lipolysis, and promotes the liberation of NEFAs [213], along with the generation of proinflammatory cytokines [214]. Increased delivery of NEFA to the liver leads to hepatic steatosis, and facilitate alcohol-induced liver injury by activating KCs [215]. Additionally, saturated NEFAs have a proinflammatory effect by activating myeloid cells, promoting cytokine release and endothelial adhesion [216]. TNFα secreted from adipose tissue can induce hepatocyte apoptosis and exacerbate inflammation through activation of the NF-κB and JNK pathways, among other pathways [217, 218].

Adipose tissue modulates homeostatic functions in both adipose and multiple non-adipose organs through the secretion of diverse bioactive molecules, collectively termed adipokines [219]. Adiponectin, the most abundant adipokine in circulation [220], exerts its biological effects via two primary receptors (AdipoR1 and AdipoR2) [221], these receptors exhibit differential expression patterns across hepatic cell populations, including hepatocytes, HSCs, and KCs, mediating beneficial effects such as anti-inflammatory responses and anti-fibrotic actions [222, 223]. Chronic inflammation of adipose tissue caused by alcohol inhibits the release of adiponectin, which in turn impairs lipid metabolism in the liver and exacerbates the progression of injury [224]. In contrast, elevated serum leptin levels in alcoholic cirrhosis demonstrate a positive correlation with the severity of hepatic pathology [225]. Alcohol abuse-induced hyperleptinemia exacerbates hepatic fibrosis by promoting HSC activation [226]. Furthermore, leptin potentiates hepatic inflammation through TNF-α release from Kupffer cells and stimulates CCL2 production in HSCs [227].

### Gut-brain-liver axis

The central nervous system (CNS) represents another critical target of alcohol-induced toxicity and neurodegeneration. Ethanol exerts dual pathological effects through direct neurotoxic actions and activation of neuroimmune signaling pathways, driving alcohol abuse-associated neuroinflammation, neuronal apoptosis, cerebral functional impairment, and addictive behaviors [3]. While abstinence remains the cornerstone intervention for alcohol-associated liver disease (ALD), the refractory nature and high relapse propensity of AUD necessitate deeper investigations into fundamental regulatory mechanisms [14].

Emerging evidence highlights bidirectional communication between the gut microbiota and the CNS via neuroendocrine and neuroimmune pathways, collectively termed the gut-brain axis [228]. Dysregulation of the gut microbiota has been linked to alcohol addiction, withdrawal symptoms, and inflammatory responses [229–231]. Day et al. demonstrated a significant correlation between intestinal *Candida albicans* colonization density and alcohol consumption patterns in murine models, providing experimental validation for microbial involvement in AUD pathogenesis [232]. Notably, sodium valerate (a microbiota-derived metabolite structurally analogous to γ-aminobutyric acid (GABA)), has been identified as a novel therapeutic candidate. This compound modulates gut microbial composition, reduces microbial-derived neuroactive metabolites through GABA degradation pathways, and attenuates excessive alcohol intake and anxiety-like/approach-avoidance behaviors in male mice [233]. These findings position probiotic-mediated microbial homeostasis restoration as a promising strategy for AUD management.

Beyond AUD specific interventions, alcohol exposure disrupts circadian rhythm regulation by altering mRNA expression of clock genes in the suprachiasmatic nucleus. Intriguingly, non-invasive 40 Hz light flicker exposure activates hypothalamic SIRT1 expression, mitigating ethanol-induced hepatotoxicity and hepatic steatosis through central clock modulation [234]. Blockade of IL-17 signaling pathway effectively reverses alcohol-associated hepatic injury and compulsive drinking behaviors in dependent mice [140, 235], suggesting therapeutic potential of inflammatory cytokine targeting along the brain-liver axis. The hepatic vagus nerve, a critical component of the nervous system, intricately innervates the liver and plays an essential role in liver-brain interactions [236]. The nervous system precisely discharges acetylcholine from the terminals of the vagus nerve [236]. This neurotransmitter acts by inhibiting TNFα synthesis via the α7 nicotinic acetylcholine receptors (α7nAChRs) present on the surface of hepatic KCs, demonstrating neural regulation of hepatic inflammation [237]. These mechanistic insights underscore the importance of understanding alcohol’s multiorgan pathological network for developing comprehensive therapeutic strategies against ALD [15, 219].

## Advances in targeting inflammation for the treatment of alcohol-associated liver disease

**Fig. 3:**
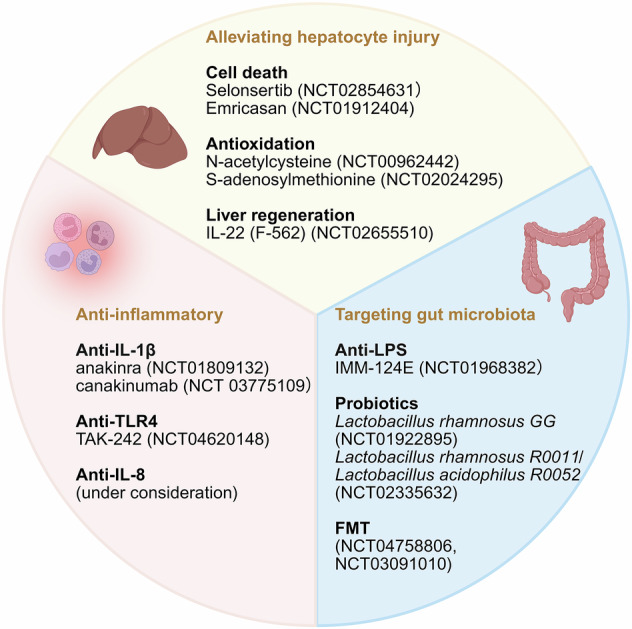
Emerging Therapeutic Targets and Clinical Trials for ALD. Three main therapeutic strategies are currently being explored: (1) Protecting hepatocytes by reducing apoptosis, alleviating oxidative stress, and promoting liver regeneration; (2) Targeting inflammatory pathways to mitigate liver and systemic inflammation; and (3) Modulating the gut-liver axis through strategies such as lowering LPS levels, probiotic supplementation, and fecal microbiota transplantation. These approaches aim to address key mechanisms driving ALD progression and improve clinical outcomes

Existing effective interventions for ALD include nutritional support, alcohol abstinence therapy, hormonal treatment, and liver transplantation [238–240]. Additionally, medications such as naltrexone, and acamprosate have proven efficacy in AUD [241, 242]. These drugs modulate opioid pathways within the CNS [243]. Corticosteroids can improve short-term survival rates, typically around 28 days, but do not enhance long-term survival rates, which are usually measured at 6 months [244]. Additionally, corticosteroids may lead to concurrent infections [244]. Pentoxifylline, a selective phosphodiesterase inhibitor, can reduce pro-inflammatory cytokine levels and is considered a potential therapeutic option for AH [245]. However, clinical trials have shown that pentoxifylline is not effective in improving the survival rate of AH patients [246]. The following section summarizes various therapeutic targets that have been clinically tested, with a focus on targeting inflammation, and discusses potential anti-inflammatory strategies for treating ALD (Fig. 3).

### Targeting inflammasomes

Therapies targeting inflammasomes are a subject of extensive research. Since the inhibition of ASK1 (apoptosis signal-regulation kinase 1) in NLRP3 (NOD-like receptor thermal protein domain associated protein 3) mutant mice can reduce liver fibrosis, hepatocyte death, and liver TNF-α expression, it suggests the application prospects of ASK1 inhibitor selonsertib in liver diseases. A phase 2 clinical trial, conducted in a double-blind, placebo-controlled manner, aimed to assess the safety and efficacy of selonsertib in combination with prednisolone against a placebo paired with prednisolone (NCT02854631). The study revealed no significant differences in the Lille response on day 7, alterations in the MELD score on day 28, or mortality rates on day 28 between the treatment and control groups [247]. The overall infection rate did not significantly differ between the groups. Caspase 1 is known to trigger inflammatory responses, whereas caspase 8 is implicated in regulating apoptosis and subsequent necrosis across various hepatic conditions. The pan-caspase inhibitor emricasan has been under investigation for patients with severe AH (NCT01912404), but the trial was halted prematurely due to concerns regarding suboptimal drug bioavailability, which led to a reduction in the safe dosage.

### Antioxidant therapies

N-acetylcysteine (NAC), an antioxidant agent, has the capacity to replenish glutathione levels in hepatocytes and alleviate oxidative stress-induced liver damage [248–250]. However, NAC treatment did not provide survival benefits at the 6-month mark for patients with severe AH compared with a placebo (NCT00962442) [250]. Furthermore, when NAC was used as an adjunct to prednisolone, it did not increase the 6-month survival rate compared with prednisolone monotherapy (NCT00863785) [249]. S-adenosylmethionine (SAMe), which serves as a direct precursor to glutathione, is currently being evaluated for its safety, efficacy, and effectiveness in an ongoing phase 4 randomized clinical trial (NCT02024295) for patients with AH and patients with alcohol-associated cirrhosis (NCT04250259).

### Targeting LPS

LPS is a key inducer of inflammatory responses [251]. Studies have shown that IMM-124E is safe for use in patients with severe AH, but it does not reduce circulating LPS levels or mortality (NCT01968382). TAK-242, a TLR4 inhibitor, is currently being tested in an RCT for alcohol-associated cirrhosis and ACLF (NCT04620148) [252]. Combining LPS neutralization with inhibitors of downstream signaling pathways may represent a promising approach for treating ALD.

### Targeting IL-1, IL-22, and chemokine pathways

Despite evidence that IL-1 significantly contributes to the progression of ALD in experimental models, clinical trials using IL-1 receptor antagonists [253] (anakinra, NCT01809132) and monoclonal antibodies [254] (canakinumab, NCT03775109) for severe AH have not demonstrated positive outcomes. In preclinical ALD models, the dual chemokine receptor CCR2/CCR5 antagonist cenicriviroc has shown promise in preventing macrophage infiltration and protecting the liver from inflammatory damage [255].

Preclinical studies indicate that IL-22 effectively mitigates liver injury associated with ALD [256, 257]. F-652, a recombinant fusion protein composed of human IL-22 and IgG2-Fc, targets IL-22R1 on epithelial cells, exerting minimal effects on immune cells and protecting tissues from damage and inflammation while promoting tissue repair [258]. An open-label phase II study of F-652 showed significant improvement in MELD scores for 18 patients by day 4 [259]. Further clinical trials are needed to confirm the benefit of F-652 in severe AH. The autocrine IL-8 loop enhances neutrophil recruitment and activation in severe AH, sustaining liver inflammation through p38 MAPK activation [75]. Thus, targeting IL-8 or CXCR1/2 holds promise as a treatment strategy for sAH [85]. Overall, therapies targeting cytokines or chemokines show potential for ALD treatment but remain under investigation. Additional research is required to establish their efficacy, safety, and to develop more effective treatment strategies.

### Gut microbiota modulation

Probiotics and prebiotics are used to correct the imbalance of the gut microbiota in alcohol-induced liver damage [207]. Recent animal studies have shown that strains such as *Lactobacillus plantarum* and *Lactobacillus rhamnosus GG* can alleviate alcohol-associated hepatic steatosis, liver injury, and gut microbiota dysbiosis [194, 260, 261]. A diet high in soluble fiber has also been found to reduce alcohol-induced liver injury by increasing the abundance of *Bacteroides acidifaciens* [211]. A randomized, placebo-controlled study demonstrated that 1 month of oral *Lactobacillus rhamnosus GG* significantly reduced liver injury in patients with ALD (NCT01922895) [239]. Another double-controlled randomized prospective clinical trial is ongoing to investigate the effects of oral supplementation with *Lactobacillus rhamnosus R0011/Lactobacillus acidophilus R0052* in patients with AH (NCT02335632) [262].

Currently, several comprehensive clinical trials are investigating the efficacy of fecal microbiota transplantation (FMT) in patients with AH (e.g., NCT04758806, NCT03091010) and liver cirrhosis (e.g., NCT04932577). These trials aim to assess various aspects of FMT treatment, including efficacy, potential side effects, long-term outcomes, and the mechanisms by which FMT might exert therapeutic effects in these specific conditions [239, 262]. FMT is currently considered a safe and effective treatment method, but potential risks remain, such as infections, allergic reactions, and gastrointestinal dysfunction. Prior to FMT treatment, it is crucial to assess the patient’s condition and risk factors carefully and to select an appropriate donor and treatment plan.

## Conclusion and perspective

Inflammation plays a pivotal role in the progression of ALD, driving the pathological continuum from steatosis to fibrosis and beyond. While neutrophil infiltration is a hallmark of severe AH, the inflammatory landscape in ALD is more complex, involving macrophages, T cells, and NKT cells. These immune cells interact with hepatocytes and nonparenchymal liver cells, orchestrating an intricate inflammatory response through the secretion of various mediators [45].

However, inflammation is not inherently detrimental—it also plays a critical role in liver repair and antimicrobial defense, particularly against bacterial infections. Many inflammatory mediators, such as TNF-α, exhibit dual roles in ALD pathogenesis, contributing to liver injury and fibrosis while simultaneously supporting liver regeneration and immune defense. This complexity may, in part, explain why anti-inflammatory therapies targeting these mediators have yielded disappointing results in clinical trials. The widespread prevalence of alcohol consumption makes mitigating ALD progression particularly challenging. Despite decades of research on inflammation in AH, clinical trials of anti-inflammatory therapies have largely failed to achieve meaningful improvements in patient outcomes. The heterogeneity of inflammatory responses among AH patients likely contributes to these setbacks, underscoring the need for a more individualized approach. Fortunately, advancements in precision medicine and high-resolution inflammatory profiling are shedding new light on disease mechanisms. By leveraging these insights, the future of AH treatment may shift toward targeted, patient-specific interventions that overcome the translational research deadlock and offer new hope for effective therapies.
